# Quality of Preventive and Chronic Illness Care for Insured Adults With Opioid Use Disorder

**DOI:** 10.1001/jamanetworkopen.2021.4925

**Published:** 2021-04-08

**Authors:** Kelly E. Anderson, G. Caleb Alexander, Lauren Niles, Sarah H. Scholle, Brendan Saloner, Sydney M. Dy

**Affiliations:** 1 Department of Health Policy and Management, Johns Hopkins Bloomberg School of Public Health, Baltimore, Maryland; 2 Department of Epidemiology, Johns Hopkins Bloomberg School of Public Health, Baltimore, Maryland; 3 Center for Drug Safety and Effectiveness, Johns Hopkins Bloomberg School of Public Health, Baltimore, Maryland; 4 Division of General Internal Medicine, Johns Hopkins School of Medicine, Baltimore, Maryland; 5 OptumLabs Visiting Fellow, OptumLabs, Eden Prairie, Minnesota; 6 National Committee for Quality Assurance, Washington, DC

## Abstract

**Importance:**

Nearly all initiatives to improve care for individuals with opioid use disorder (OUD) have focused on improving OUD identification and treatment. Whether individuals with OUD have lower quality of care than individuals without OUD remains unclear.

**Objective:**

To measure quality of non-OUD preventive and chronic illness care and care coordination for individuals with OUD compared with individuals without OUD.

**Design, Setting, and Participants:**

A cross-sectional study of deidentified data on outpatients throughout the US was conducted. Claims for 79 372 commercially insured and Medicare Advantage enrollees aged 18 years or older with diagnosis codes for OUD between January 1, 2018, and December 31, 2019, and 46 601 individuals without OUD were included in the analysis.

**Exposure:**

Diagnosis of OUD.

**Main Outcomes and Measures:**

Quality indicator performance was calculated, using claims for individuals with OUD and matched comparators without OUD. Within 3 domains of outpatient care quality (preventive care, chronic illness care, and care coordination), 6 indicators used in accountability programs were selected. Performance for individuals with and without OUD was compared, and logistic regression was used to analyze sociodemographic and comorbidity characteristics associated with higher quality of health care.

**Results:**

The study included 125 973 individuals, including 69 466 (55.1%) women and 78 225 (62.1%) White individuals, with a mean (SD) age of 59.0 (16.1) years. For the preventive care measure examining breast cancer screening, performance for the OUD cohort was 55.4% (95% CI, 54.7%-56.0%) compared with 65.6% (95% CI, 64.4%-66.7%) for individuals without OUD (*P* < .001). Quality of care for adherence to statin therapy was lower for individuals with OUD (70.4%; 95% CI, 68.7%-72.1%) compared with individuals without OUD (76.7%; 95% CI, 74.4%-78.7%) (*P* < .001) and for the hemoglobin A_1c_ testing indicator (OUD: 80.9%; 95% CI, 80.4%-81.5%; comparator: 85.8%; 95% CI, 84.9%-86.8%; *P* < .001). Care coordination quality also was lower for individuals with OUD compared with those without OUD for mental health follow-up (OUD: 45.3%; 95% CI, 44.6%-46.0%; comparator: 52.5%; 95% CI, 50.0%-55.0%; *P* < .001) and for potentially avoidable hospitalizations for chronic conditions (OUD: 11.4%; 95% CI, 11.2%-11.7%; comparator: 8.8%; 95% CI, 8.3%-9.2%; *P* < .001) and diabetes, where a lower score indicates higher quality (OUD: 2.4%; 95% CI, 2.3%-2.5%; comparator: 1.9%; 95% CI, 1.7%-2.1%; *P* = .001).

**Conclusions and Relevance:**

These findings suggest that individuals with OUD have moderately lower quality of care across preventive and chronic illness care and care coordination for non-OUD care compared with individuals without OUD. More attention to measurement and improvement of non-OUD care for these individuals is needed.

## Key Points

**Question:**

What is the quality of care for health needs other than treatment of opioid use disorder in individuals with opioid use disorder compared with those without opioid use disorder?

**Findings:**

In this cross-sectional study of 79 372 insured adults with opioid use disorder and a comparison group without opioid use disorder, quality of care was between 0.5 and 10.2 percentage points lower for those with opioid use disorder for preventive care, chronic illness care, and care coordination. All of these differences were statistically significant.

**Meaning:**

The findings of this study suggest that more attention to measurement and improvement of preventive and chronic illness care for needs other than treatment of opioid use disorder for these individuals is needed.

## Teaser

This cross-sectional study examines the quality of care for conditions other than opioid use disorder in individuals with opioid use disorder.

## Introduction

In 2018, nearly 70 000 individuals in the US died from drug overdoses, with more than two-thirds of the deaths linked to opioids.^1^ An estimated 1.6 million individuals in the US had active opioid use disorder (OUD) in 2019.^2^

Given the high morbidity and mortality associated with OUD, health systems, payers, and policy makers have invested substantial financial and regulatory attention to improving access to OUD treatment.^3^ Although studies have begun to evaluate quality of OUD care,^4^ limited research has examined the quality of care that individuals with OUD receive for non-OUD conditions. This topic is important because chronic conditions common among the general population, such as hypertension, hyperlipidemia, and diabetes, are also common among individuals with OUD, and OUD is associated with higher odds of having conditions such as diabetes and heart disease.^5,6,7,8,9^ In addition, because OUD is stigmatized and associated with significant impairment, the quality of preventive care and treatment of other conditions could be lower, contributing to poorer health.^10,11,12^ Expanding provision of buprenorphine and links to comprehensive OUD treatment in primary care should facilitate addressing preventive care and chronic conditions, and numerous organizations have recommended this improved integration of care.^7,10,13,14,15,16^ Sociodemographic and comorbidity characteristics could also be associated with disparities in non-OUD quality of care. Similarly, research examining quality of medical care for individuals with serious mental illness has reported substantial gaps in quality indicator performance compared with the general population.^17^ For individuals with serious mental illness, such gaps in care contribute to premature mortality, and it is important to understand whether similar gaps in quality of care exist for individuals with OUD.

We compared the quality of care of insured adults with OUD for non-OUD care with a comparison group of similar (propensity-score matched) insured adults to examine whether individuals with OUD have lower quality of care. We conducted this study using a large, diverse database of commercially insured and Medicare Advantage enrollees in the US. We focused on 6 quality indicators from well-established programs, such as those from the Centers for Medicare & Medicaid Services,^13^ spanning preventive care, chronic illness care, and care coordination. We quantified quality of care among those with OUD and compared it with individuals without OUD and explored whether individuals’ sociodemographic and comorbidity characteristics were associated with the quality of care received.

## Methods

### Data Source

We performed a cross-sectional study of data from the OptumLabs Data Warehouse, which includes deidentified claims data for commercially insured and Medicare Advantage enrollees from large health plans.^18^ The database contains sociodemographic and longitudinal information regarding inpatient, outpatient, and prescription drug use, representing a diverse mixture of ages, race/ethnicities, and geographic regions across the US (eAppendix in the Supplement). In addition to other characteristics, we examined group differences based on race/ethnicity, because structural racism often leads to worse quality of care.^19^ The OptumLabs Data Warehouse identifies race/ethnicity from a national supplier of consumer marketing data that imputes race and ethnicity using an individual’s name and geographic location (eAppendix in the Supplement). Other sociodemographic characteristics, including imputed household income and median census tract educational level, are also sourced from this national supplier of consumer marketing data.

Our study was exempt from Johns Hopkins Bloomberg School of Public Health Institutional Review Board review and informed consent because the data are deidentified and the study was therefore not considered human participants research. This study followed the Strengthening the Reporting of Observational Studies in Epidemiology (STROBE) reporting guideline for cross-sectional studies.

Using the OptumLabs Data Warehouse, there are 2 primary ways in which missing data may affect our study. First, if individuals lose or switch health insurance coverage during the period of analysis, we may not accurately classify the quality of their care. This possibility is small because we used a stringent method for defining our cohort to ensure that we had a relatively complete picture of a full year of an individual’s care. Second, we may be missing sociodemographic information for individuals included in our study. We report the extent of missing data for sociodemographic variables.

### Derivation of Cohorts

We identified 164 232 individuals with an OUD diagnosis (index diagnosis) based on *International Statistical Classification of Diseases, 10th Revision* codes in the inpatient, outpatient, or emergency department setting who had at least some commercial or Medicare Advantage coverage between January 1, 2018, and December 31, 2019 (eFigure 1 and eTable 1 in the Supplement). We then excluded (1) 62 676 individuals with incomplete medical or pharmacy coverage during the 90 days before and 12 months after their index diagnosis, (2) 11 587 individuals with a diagnosis of OUD during a 90-day washout period before their index date, (3) 10 205 individuals with an outpatient index diagnosis without a subsequent outpatient OUD claim,^14,20^ and (4) 392 individuals younger than 18 years. Our final analytic sample included 79 372 individuals with OUD.

We compared quality of care of individuals with OUD with a comparison group drawn from a random sample of individuals without OUD and matched to those with OUD based on sociodemographic and comorbidity characteristics. To derive the comparison group, we drew a random sample of 1 million individuals with at least some 2018 or 2019 commercial or Medicare Advantage coverage without any diagnosis of OUD or claim for US Food and Drug Administration–approved medications to treat OUD (buprenorphine, naltrexone, or methadone) during the study period. We then excluded 380 513 individuals with incomplete medical and pharmacy coverage during the study period; 150 350 individuals who did not have at least 1 claim in the inpatient, 2 claims in the outpatient, or 1 claim in the emergency department setting in their index year (to mimic the use requirement to identify individuals with OUD); and 72 782 individuals younger than 18 years during their index year. From the remaining 396 355 individuals, we conducted 1:1 nearest neighbor propensity score matching with replacement^21^ in R version 3.6.1, using the MatchIt package.^22^ Propensity score matching allowed us to identify a subset of individuals without OUD with similar sociodemographic and comorbidity characteristics as the OUD cohort. We completed propensity score matching separately for individuals with commercial and Medicare Advantage coverage to improve the quality of the match and allow stratifying analyses by insurance type while ensuring that each OUD case had a matching non-OUD comparison. The full list of sociodemographic and comorbidity characteristics used for propensity score matching is provided in eFigure 2 in the Supplement.

### Selection of Quality Indicators

We selected quality indicators using several criteria. First, we searched for indicators in 3 core domains commonly used in quality programs: preventive care, chronic illness care, and care coordination. We next limited identified indicators to those specified to use administrative and pharmacy claims data and used in accountability programs in 2020, such as Centers for Medicare & Medicaid Services programs (Accountable Care Organizations,^13^ Merit-Based Incentive Payment System,^23^ and Medicare Star Ratings^24^), the Medicaid Core Sets,^25^ or the Healthcare Effectiveness Data and Information Set for health plans.^26^ We made final determinations on inclusion if the indicator could be successfully implemented in OptumLabs claims data and reached a denominator of 2000 individuals as sufficiently common to be considered in population quality initiatives. The final list included 1 preventive care indicator, 2 chronic illness care indicators, and 3 care coordination indicators (Table 1).^13^

**Table 1.** Selected Quality Indicators

Indicator category	Indicator	Description
Prevention	Breast cancer screening	Percentage of women aged 50-74 y in the analytic sample who had ≥1 mammogram to screen for breast cancer in the past 2 y
Chronic disease management	Statin adherence for patients with cardiovascular disease	Percentage of men aged 21-75 y and women aged 40-75 y with atherosclerotic cardiovascular disease in the analytic sample who continued receiving a statin medication for ≥80% of their treatment period
	Comprehensive diabetes control (HbA_1c_ testing)	Percentage of individuals aged 18-75 y with diabetes (type 1 and 2) in the analytic sample who had an HbA_1c_ test during the year
Care coordination	Follow-up after hospitalization or ED visit for mental illness	Percentage of hospitalizations or ED visits for treatment of mental illness or intentional self-harm (primary diagnosis) that resulted in a follow-up visit with a mental health professional within 30 d post discharge
	Potentially avoidable hospitalizations (chronic composite)^a^	Percentage of admissions for 1 of the following conditions: diabetes with short-term complications, diabetes with long-term complications, uncontrolled diabetes without complications, diabetes with lower-extremity amputation, chronic obstructive pulmonary disease, asthma, hypertension, or heart failure without a cardiac procedure
	Potentially avoidable hospitalizations (diabetes composite)	Percentage of admissions for 1 of the following conditions: diabetes with short-term complications, diabetes with long-term complications, uncontrolled diabetes without complications, diabetes with lower-extremity amputation

Abbreviations: ED, emergency department; HbA_1c_, hemoglobin A_1c_.

^a^ Potentially avoidable hospitalizations are from the Agency for Healthcare Research and Quality prevention quality indicators; these are classified as care coordination indicators as in the Accountable Care Program.^10^

### Statistical Analysis

We first compared sociodemographic and comorbidity characteristics of individuals in the OUD cohort with the matched comparison cohort. We summarized the presence of comorbid conditions using the Elixhauser Comorbidity Index^27^; scores range from 0 to 30, with a higher score indicating more comorbid conditions. We excluded the drug use disorder category from the index score calculation. Next, we calculated the performance of each group on each indicator. To do so, we used the quality indicator specifications described in Table 1 to identify the individuals eligible for the indicator (measure denominator) and determine whether each eligible individual met the numerator criteria for the indicator. We aggregated performance for all eligible individuals in each cohort to compare quality of care for each of the indicators for individuals with OUD and the matched comparison group without OUD. For individuals with OUD, we then compared performance based on insurance coverage to examine how quality of care for individuals with OUD differed depending on whether they had Medicare Advantage or commercial coverage. We then used multivariable logistic regressions to estimate adjusted differences in quality of care and calculated estimated probabilities of receiving high-quality care based on sex, insurance type, race/ethnicity, educational level, income, Elixhauser Comorbidity Index score, and comorbid conditions (eTable 2 in the Supplement). We ran separate regressions with each quality indicator as the dependent variable for individuals with OUD and the matched comparison group of individuals without OUD. From the regressions, we calculated mean predicted probabilities of receiving high-quality care. We considered differences of greater than 5 percentage points in quality to be moderate quality gaps.^28^

We extracted data using structured query language, conducted propensity score matching in R version 3.6.1, and completed all analyses using Stata version 15 (StataCorp). Findings were considered significant at 2-tailed *P* < .05.

## Results

The study sample comprised 125 973 individuals, including 69 466 (55.1%) women, 56 507 (44.9%) men, and 78 225 (62.1%) White individuals; mean (SD) age was 59.0 (16.1) years. Table 2 depicts characteristics of the 79 372 individuals with OUD and the matched comparison group weighted to represent 79 372 individuals without OUD. Standardized differences for the OUD and matched samples were less than 0.1 SD for all sociodemographic and comorbidity characteristics used for matching (Table 2; eFigure 2 in the Supplement).

**Table 2.** Characteristics of Study Cohort

Characteristic	No. (%)	
	Individuals with OUD (n = 79 372)	Matched comparators without OUD (n = 79 372)^a^
Sex		
Women	43 904 (55.3)	44 181 (55.7)
Men	35 468 (44.7)	35 191 (44.3)
Medicare Advantage coverage	54 297 (68.4)	54 297 (68.4)
Age, y		
18-34	7122 (9.0)	6753 (8.5)
35-49	12 396 (15.6)	12 361 (15.6)
50-64	27 886 (35.1)	28 266 (35.6)
65-74	20 141 (25.4)	19 976 (25.2)
≥75	11 827 (14.9)	12 016 (15.1)
Race/ethnicity		
Non-Hispanic		
White	49 230 (62.0)	49 471 (62.3)
Black	9303 (11.7)	9412 (11.9)
Hispanic	6162 (7.9)	6099 (7.7)
Asian	660 (0.8)	504 (0.6)
Unknown	14 017 (17.7)	13 886 (17.5)
Educational level		
≤High school	26 290 (33.1)	26 846 (33.8)
Some college	34 509 (43.5)	34 432 (43.3)
≥Bachelor’s degree	6319 (8.0)	5683 (7.2)
Missing/unknown	12 254 (15.4)	12 411 (15.6)
Household income, $		
<40 000	22 526 (28.4)	22 890 (28.8)
40 000-74 999	16 698 (21.0)	16 961 (21.4)
75 000-124 999	12 976 (16.3)	12 803 (16.1)
125 000-199 000	4841 (6.1)	4544 (5.7)
≥200 000	2388 (3.0)	2137 (2.7)
Missing/unknown	19 943 (25.1)	20 037 (25.2)
Elixhauser Comorbidity Index^b^		
0	5449 (6.9)	5422 (6.8)
Low (1-2)	18 379 (23.2)	18 251 (23.0)
Medium (3-6)	32 951 (41.5)	32 889 (41.4)
High (≥7)	22 593 (28.5)	22 810 (28.7)
Elixhauser Comorbidity Index, mean^b^	4.9	4.7
Chronic conditions		
Hypertension	53 709 (67.7)	54 028 (68.1)
Hyperlipidemia	43 590 (54.9)	47 403 (59.7)
CAD	15 209 (19.2)	15 202 (19.2)
CVA	2611 (3.3)	2588 (3.3)
Asthma	10 419 (13.1)	10 900 (13.7)
COPD	22 888 (28.8)	18 794 (23.7)
Arthritis	47 390 (59.7)	38 260 (48.2)
Diabetes	24 777 (31.2)	27 337 (34.4)
Depression	36 112 (45.5)	35 947 (45.3)
Anxiety disorder	36 631 (46.2)	36 445 (45.9)
Bipolar disorder	9079 (11.4)	8173 (10.3)
Hepatitis C	3271 (4.1)	1291 (1.6)
HIV/AIDS	548 (0.7)	463 (0.6)
Alcohol use disorder	8834 (11.1)	7784 (9.8)
Chronic pain	55 585 (70.0)	56 251 (70.9)

Abbreviations, CAD, coronary artery disease; COPD, chronic obstructive pulmonary disease; CVA, cerebrovascular accident; OUD, opioid use disorder.

^a^ The OUD cohort was matched to comparators using 1:1 nearest neighbor matching with replacement. Using propensity score weights, the 46 601 matched comparators simulate 79 372 individuals. Weighted estimates of sociodemographic and comorbidity characteristics are presented here.

^b^ Elixhauser Comorbidity Index scores range from 0 to 30, with a higher score indicating a greater number of comorbid conditions. We excluded the drug abuse category from the index score calculation.

**Figure 1.**
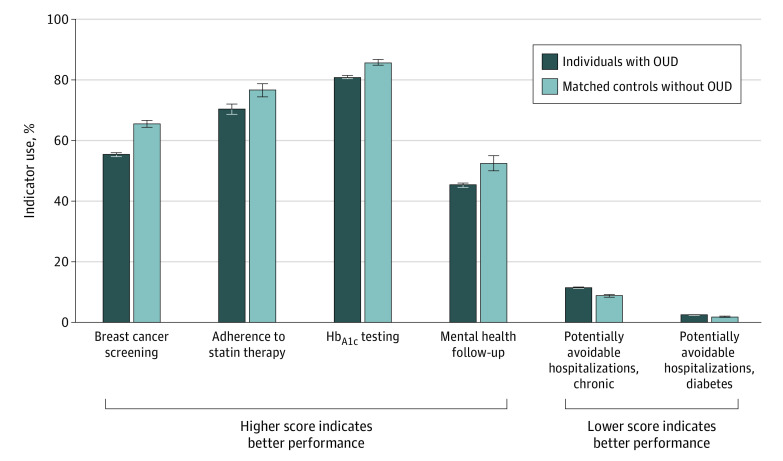
Comparison of Quality Indicator Performance for Individuals With and Without Opioid Use Disorder (OUD), 2018 to 2019 For the breast cancer screening indicator, 22 217 women with OUD and 23 083 matched comparators without OUD were eligible. For the adherence to statin therapy indicator, 2832 individuals with OUD and 5774 matched comparators without OUD were eligible. For the hemoglobin A_1c_ (HbA_1c_) testing indicator, 19 475 individuals with OUD and 21 968 matched comparators without OUD were eligible. For the mental health follow-up indicator, 18 145 individuals with OUD and 9833 matched comparators without OUD were eligible. For the chronic composite and diabetes composite indicators, 79 372 individuals with OUD and 79 372 matched comparators without OUD were eligible. The OUD cohort was matched to the control cohort using 1:1 nearest neighbor matching with replacement. Using propensity score weights, the 46 601 matched comparators simulated 79 372 individuals. This figure reports weighted estimates of quality indicator performance for the matched comparators. Error bars indicate 95% CIs.

We found a moderate difference for the prevention indicator for breast cancer screening (mammography). Quality of care was statistically significantly lower in the OUD cohort, with 12 305 of 22 217 eligible women (55.4%; 95% CI, 54.7%-56.0%) receiving high-quality care compared with 15 135 of 23 083 eligible women (65.6%; 95% CI, 64.4%-66.7%) in the matched comparison cohort without OUD (*P* < .001) (Figure 1).

We found moderate differences for chronic illness care in individuals with chronic condition indicators. Quality of care for adherence to statin therapy for coronary artery disease was significantly lower for individuals with OUD, with 1994 of 2832 eligible individuals receiving high-quality care (70.4%; 95% CI, 68.7%-72.1%) compared with 4426 of 5774 eligible individuals in the matched comparison group (76.7%; 95% CI, 74.4%-78.7%) (*P* < .001). For the hemoglobin A_1c_ testing indicator, quality of care was significantly lower for individuals with OUD, with 15 760 of 19 475 eligible individuals (80.9%; 95% CI, 80.4%-81.5%) receiving high-quality care compared with 18 857 of 21 968 eligible matched comparators (85.8%; 95% CI, 84.9%-86.8%) (*P* < .001).

We identified small to moderate differences for the care coordination indicators. The 30-day follow-up rate after an emergency department visit or inpatient hospitalization for a mental health condition was statistically significantly lower for individuals with OUD, with 8223 of 18 145 eligible individuals (45.3%; 95% CI, 44.6%-46.0%) receiving high-quality care compared with 5165 of 9833 eligible matched comparators (52.5%; 95% CI, 50.0%-55.0%) (*P* < .001). For the potentially avoidable hospitalizations chronic conditions indicator, all 79 372 individuals with OUD were eligible, and quality of care was significantly lower for those with OUD, with 9080 having had a potentially avoidable hospitalization (11.4%; 95% CI, 11.2%-11.7%) compared with 6967 of 79 372 matched comparators (8.8%; 95% CI, 8.3%-9.2%), a 2.6 percentage point (22.8%) difference (*P* < .001). For the indicator of potentially avoidable hospitalizations for diabetes, all 79 372 individuals with OUD were eligible, and quality of care was significantly lower for those with OUD, with 1878 having had a potentially avoidable hospitalization (2.4%; 95% CI, 2.3%-2.5%) compared with 1513 of 79 327 matched comparators (1.9%; 95% CI, 1.7%-2.1%), a 0.5 percentage point (20.8%) difference (*P* = .001).

**Figure 2.**
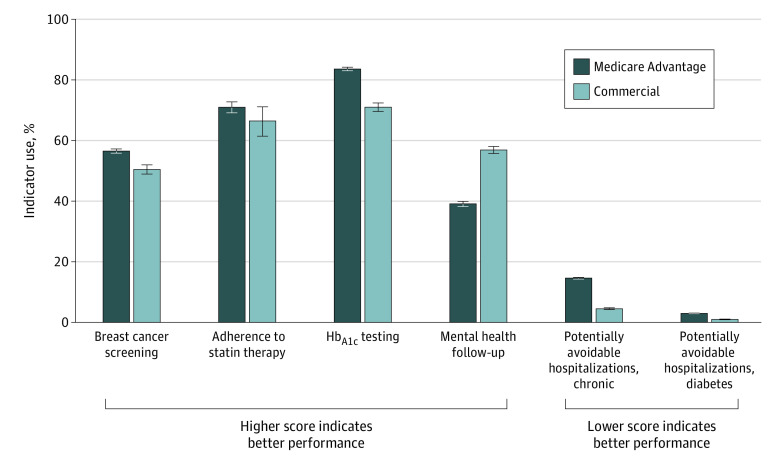
Quality Indicator Performance for Individuals With Opioid Use Disorder (OUD) With Medicare Advantage vs Commercial Insurance (2018-2019) For the breast cancer screening indicator, 17 772 individuals with Medicare Advantage coverage and 4445 individuals with commercial coverage were eligible. For the adherence to statin therapy indicator, 2453 individuals with Medicare Advantage coverage and 379 individuals with commercial insurance were eligible. For the hemoglobin A_1c_ (HbA_1c_) testing indicator, 15 290 individuals with Medicare Advantage coverage and 4185 individuals with commercial insurance were eligible. For the mental health follow-up indicator, 11 776 individuals with Medicare Advantage coverage and 6369 individuals with commercial insurance were eligible. For the chronic composite and diabetes composite potentially avoidable hospitalization indicators, 54 297 individuals with Medicare Advantage coverage and 25 075 individuals with commercial insurance were eligible. Error bars indicate 95% CIs.

Among individuals with OUD, for preventive care (breast cancer screening), quality of care was statistically significantly better for eligible individuals with Medicare Advantage (10 060 of 17 772 [56.6%; 95% CI, 55.9%-57.3%]) compared with commercial insurance (2245 of 4445 [50.5%; 95% CI, 49.0%-52.0%]) (*P* < .001). Similarly, for chronic illness care, performance on the HbA_1c_ testing indicator was significantly better for individuals with Medicare Advantage (12 789 of 15 290 [83.6%; 95% CI, 83.0%-84.2%]) than commercial insurance (2971 of 4185 [71.0%; 95% CI, 69.6%-72.4%]). In contrast, for coordination of care, quality of care was worse with Medicare Advantage for all 3 indicators; for example, for the indicator of follow-up following an emergency department visit or inpatient hospitalization for mental health, the indicator score was 17.8 percentage points lower for individuals with Medicare Advantage (4600 of 11 776 [39.1%; 95% CI, 38.2%-40.0%]) compared with commercial insurance (3623 of 6369 [56.9%; 95% CI, 55.7%-58.1%]) (Figure 2).

In general, quality indicator performance did not meaningfully differ by sociodemographic or comorbidity characteristics; patterns were similar between those with OUD and matched patients without OUD based on the calculated predicted probability of meeting each quality indicator’s numerator criteria. The most common differences were noted for individuals with lower income (eg, breast cancer screening in those with OUD: 54.2% for those with an income <$40 000 to 64.4% for those with an income >$200 000 annually) and for lower comorbidity (eg, breast cancer screening in those with OUD: 46.6% for those with no comorbidities to 55.9% for those with high Elixhauser Comorbidity Index scores (≥7; *P* < .001 for both). Predicted probabilities for key variables are reported in Table 3; full results are presented in eTable 2 in the Supplement.

**Table 3.** Estimated Probabilities of Quality Indicator Performance by Sociodemographic and Comorbidity Characteristics for OUD vs Non-OUD Comparators^a^

Characteristic^b^	Estimated probability, %^c^											
	Breast cancer		Statin adherence		HbA_1c_ testing		Mental health follow-up		PAH			
									Chronic		Diabetes	
	OUD (n = 22 217)	Matched comparators (n = 25 919)	OUD (n = 2825)	Matched comparators (n = 5774)	OUD (n = 19 475)	Matched comparators (n = 21 968)	OUD (n = 18 145)	Matched comparators (n = 9833)	OUD (n = 79 372)	Matched comparators (n = 79 372)	OUD (n = 79 372)	Matched comparators (n = 79 372)
Sex												
Men, reference group^d^	NA	NA	70.5	77.2	81.1	85.3	44.5	46.6	12.8	10.0	9.3	6.6
Women	55.4	64.5	70.1	76.0	80.8	86.3	46.0^e^	56.3^e^	11.9^e^	9.0^f^	6.9^e^	5.2^f^
Insurance type												
Commercial, reference group	50.5	66.8	63.7	72.3	79.3	85.0	50.9	59.1	13.3	9.6	11.6	7.7
Medicare Advantage	56.6^e^	64.1^f^	71.3^e^	77.0	81.4^e^	86.1	42.3^e^	48.9^e^	12.1^e^	9.4	7.5^e^	5.6^f^
Race												
White, reference group	54.5	62.4	70.9	77.2	80.6	84.9	46.8	59.1	12.3	9.3	7.9	5.6
Black	58.5^e^	67.3^e^	66.0	69.0^e^	79.9	86.0	45.4	42.8^e^	12.8	11.0^e^	8.8	7.4
Asian	54.0	65.6	83.0	77.8	78.7	91.8^f^	41.0	47.2	11.8	13.2	10.6	4.6
Hispanic	62.1^e^	66.7^f^	74.2	74.3	85.9^e^	86.1	47.5	43.3^e^	10.8^e^	9.0	6.8	8.2^f^
Educational level												
≤High school diploma, reference group	56.2	65.5	68.0	78.0	80.5	85.2	39.5	50.7	12.3	9.1	7.9	5.3
Some college	56.1	66.5	70.4	75.6	81.1	86.7	45.7^e^	48.7	12.4	9.9	7.9	6.1
≥College degree	57.0	71.5^f^	75.1	79.3	81.9	85.7	51.0^e^	57.3	12.2	9.1	7.2	4.8
Income, $												
<40 000, reference group	54.2	64.8	71.6	75.0	81.3	86.5	42.9	49.8	12.4.	9.9	8.0	6.2
40 000-74 999	59.3^e^	67.5	69.9	77.9	81.1	85.6	46.8^e^	53.2	11.8	8.9	7.1^f^	4.9
75 000-124 999	59.7^e^	69.3^f^	73.9	80.2	81.9	86.5	53.6^e^	55.6	11.9	8.9	6.8^f^	5.9
125 000-199 999	60.6^e^	68.0	72.1	71.0	80.3	85.9	50.6^e^	60.5^f^	12.0	9.8	8.0	5.4
≥200 000	64.4^e^	73.1^f^	68.8	83.4	84.0	89.1	55.8^e^	73.9^e^	13.5	8.6	8.8	1.4^f^
Elixhauser Comorbidity Index												
0, reference group	46.6	62.0		91.3	60.3	79.8	21.2	32.1	NA	NA	NA	NA
Low (1-2)	52.4^e^	64.6	68.2^g^	78.3	78.0^e^	84.3	54.1^e^	65.2^e^	1.5^g^	0.7^g^	NA	NA
Medium (3-6)	56.9^e^	65.4	72.1	79.6	80.9^e^	85.5	49.6^e^	55.2	6.3^e^	4.1^e^	2.0^h^	1.4^h^
High (≥7)	55.9^e^	63.6	69.1	74.8	82.0^e^	86.7	39.1^f^	47.1	22.0^e^	17.4^d^	11.8^e^	8.8^e^

Abbreviations: HbA_1c_, hemoglobin A_1c_; NA, not applicable; OUD, opioid use disorder; PAH, potentially avoidable hospitalization.

^a^ The model used to calculate estimated probabilities included the variables noted, as well as the comorbid conditions included in Table 1. Predicted probabilities for all of the variables are reported in eTable 2 in the Supplement.

^b^ Unknown response options for race, educational level, and income were included in the model but not reported in the table.

^c^ For the PAH indicators, a lower indicator score indicates better quality of care. For all other indicators, a higher score indicates better quality of care.

^d^ Men were not eligible for the breast cancer measure.

^e^ *P* ≤ .01; significant difference between the reference group and the comparator category.

^f^ *P* ≤ .05; significant difference between the reference group and the comparator category.

^g^ Because an Elixhauser Comorbidity Index score of 0 was omitted from the chronic illness care composite indicator regression, we treated the category of low (1-2) as the reference group.

^h^ Because an Elixhauser Comorbidity Index score of 0 and a low Elixhauser Comorbidity Index score (1-2) were omitted from the chronic composite indicator regression, we treated the category of medium (3-6) as the reference group.

## Discussion

We found that insured adults with OUD had small to moderate statistically significantly lower quality of care on all 6 quality indicators across the domains of preventive care, chronic illness care, and care coordination compared with the matched sample of insured adults without OUD. Comparing individuals with Medicare Advantage with those with commercial insurance, quality of care was better for preventive and chronic illness care, but worse for care coordination. Quality of care was generally not associated with sociodemographic and comorbidity factors; lower income and fewer comorbidities were most commonly associated with lower quality of care for those with OUD.

Our work, which suggests policy-relevant quality of care differences among insured adults with OUD compared with their counterparts,^28^ highlights gaps in comprehensive care for those with OUD, including attention to preventive and chronic illness care needs. Opioid use disorder is often characterized by dysregulation,^10^ diminished self-efficacy,^11^ less future orientation,^12^ and mental health comorbidity,^8^ and this population is therefore at particular risk of preventable conditions and complications; integration of treatment for OUD with higher-quality care in these individuals may be particularly beneficial, especially given recent findings that, in Medicaid primary care, clinicians provide equal or better quality buprenorphine treatment compared with behavioral health specialists, pain specialists, and other specialty clinicians.^4^ Opioid use disorder treatment requiring engagement with primary care may increase health care contacts that facilitate improved care for other needs. A Canadian study including individuals receiving social assistance also found lower quality of care for preventive and chronic illness care in those with OUD and that primary care medical home enrollment was associated with better quality of care for some measures.^29^ Although some current models of OUD care integrate with medical home or other primary care models,^30^ and these could be associated with better quality for non-OUD conditions, an association with non-OUD outcomes has not been addressed. Ideally, the move toward more integrated primary care models, such as Primary Care First,^31^ that include behavioral health and move toward a focus on value and quality, can provide opportunities to address patients’ mental and physical health comorbidities in the context of their OUD care.

The variability in whether commercial vs Medicare Advantage coverage was associated with higher quality of care for individuals with OUD may reflect differences in clinical and sociodemographic characteristics. Alternatively, the variations in quality by type of insurance among individuals with OUD may reflect differences in payment and care management models for these populations. These variations in quality further highlight the need for research on the types of care delivery and payment models that best serve individuals with OUD.

### Limitations

This study had limitations. First, these quality indicators, although representing important aspects of non-OUD care across 3 domains, reflect limited, relatively easily measurable aspects of care. Second, important dimensions of care for this population, such as patient-centeredness and cultural competence, are difficult to assess using claims data. Third, our data source, although providing a large and diverse sample, omits information needed for important questions regarding variation in quality across clinicians or geographic regions. Fourth, we used a stringent method for defining the cohort to ensure that we had a relatively complete picture of a full year of an individual’s care. This method may exclude some groups, such as those with less-stable insurance enrollment or more precarious connection to the health system. Fifth, we focused on individuals with commercial and Medicare Advantage coverage. Although this is an understudied group of individuals with OUD, the results may not be generalizable to other populations, including those with Medicaid coverage and those who are uninsured. Sixth, residual confounding may exist owing to unobserved characteristics, such as whether an individual has stable housing. Seventh, we performed multiple statistical tests. As the number of statistical comparisons increases, so does the possibility of statistically significant results due to chance.

## Conclusions

In this study, we found that individuals with OUD received consistently lower quality of care across preventive and chronic illness care and care coordination for non-OUD health care indicators. Models for improving care and access for this population for OUD should also address these other key domains of care. Future research should evaluate clinician and delivery system factors that contribute to poor quality of medical care among individuals with OUD and consider the potential association between models integrating primary care with OUD care and quality of care and outcomes.

## Notes

Supplement. **eAppendix.** Description of OptumLabs Sociodemographic Data **eFigure 1.** Flowchart of Cohort Inclusion and Exclusion Criteria **eFigure 2.** Love Plots of Covariate Balance Before and After Propensity Score Matching **eTable 1.** *ICD-10* Codes for Opioid Use Disorder (OUD) **eTable 2.** Estimated Probabilities for Quality Measure Performance for Individuals With OUD Compared to Matched Non-OUD Comparators, Including Estimated Probabilities for Clinical Covariates
