# Supplementary material for: Quality of Preventive and Chronic Illness Care for Insured Adults With Opioid Use Disorder
Source: JAMA Netw Open. 2021 Apr 8;4(4):e214925. doi: 10.1001/jamanetworkopen.2021.4925 (PMC8033422; doi:10.1001/jamanetworkopen.2021.4925)
Supplement: Supplement. — eAppendix. Description of OptumLabs Sociodemographic Data eFigure 1. Flowchart of Cohort Inclusion and Exclusion Criteria eFigure 2. Love Plots of Covariate Balance Before and After Propensity Score Matching eTable 1. ICD-10 Codes for Opioid Use Disorder (OUD) eTable 2. Estimated Probabilities for Quality Measure Performance for Individuals With OUD Compared to Matched Non-OUD Comparators, Including Estimated Probabilities for Clinical Covariates [file jamanetwopen-e214925-s001.pdf]

Supplementary Online Content

Anderson KE, Alexander GC, Niles L, Scholle SH, Saloner B, Dy SM. Quality of preventive and chronic illness care for insured adults with opioid use disorder. *JAMA Netw Open*. 2021;4(4):e214925. doi:10.1001/jamanetworkopen.2021.4925

eAppendix. Description of OptumLabs Sociodemographic Data

eFigure 1. Flowchart of Cohort Inclusion and Exclusion Criteria

eFigure 2. Love Plots of Covariate Balance Before and After Propensity Score Matching

eTable 1. *ICD-10* Codes for Opioid Use Disorder (OUD)

eTable 2. Estimated Probabilities for Quality Measure Performance for Individuals With OUD Compared to Matched Non-OUD Controls, Including Estimated Probabilities for Clinical Covariates

This supplementary material has been provided by the authors to give readers additional information about their work.

© 2021 Anderson KE et al. *JAMA Network Open.*

eAppendix. Description of OptumLabs Sociodemographic Data

The sociodemographic data available in the OptumLabs Data Warehouse (OLDW) is sourced from a national supplier of consumer marketing data, which includes consumer-specific demographic, behavioral, and lifestyle information. Much like administrative claims, the data have been collected for purposes other than research. Race is derived from ethnicity. Ethnicity is imputed based on a model run by the data supplier using an individual's name (first, last, middle) and geographic location, then categorized into five race values in OLDW: W (White), B (Black), H (Hispanic), A (Asian), and U (Unknown). The data supplier obtains education level from the US Census Bureau's American Community Survey. Education is assigned to an individual and the value represents the median level of education achieved among all residents 25 years and older within a specified census block group. All individuals within the same census block group (regardless of age) are given the same education level value. The data supplier imputes estimated household income based on a model using both public and private consumer data (credit card statements, loan amounts and loan payments). This variable is assigned at the household level where a "household" is defined as individuals with the same surname living at the same street address. All individuals within the same household will have the same household income value.

© 2021 Anderson KE et al. *JAMA Network Open.*

eFigure 1. Flowchart of Cohort Inclusion and Exclusion Criteria

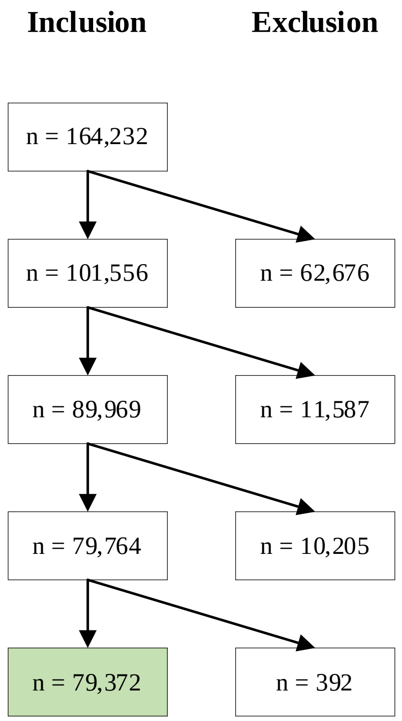

Identify: Cases with an OUD diagnosis in the inpatient or outpatient setting between 1/1/18 and 12/31/19

Continuous enrollment: Exclude cases that did not maintain continuous enrollment in medical and pharmacy coverage (gaps no longer than 45 days) in the 90 days before and 12 months after the index diagnosis

Washout period: Exclude cases with a diagnosis of OUD in the 90-day period prior to the index date

1 inpatient claim, 1 ED claim, or 2 outpatient claims: Exclude incident cases with only one outpatient claim

At least 18 years of age: Exclude patients less than 18 years of age in year of index diagnosis

Note: For individuals with a diagnosis of OUD, the index diagnosis occurred in the outpatient setting for 73.8% (56,608), in the inpatient setting for 18.3% (14,486), and in the emergency department for 7.9% (6,278) of the 79,372 individuals meeting the inclusion criteria.

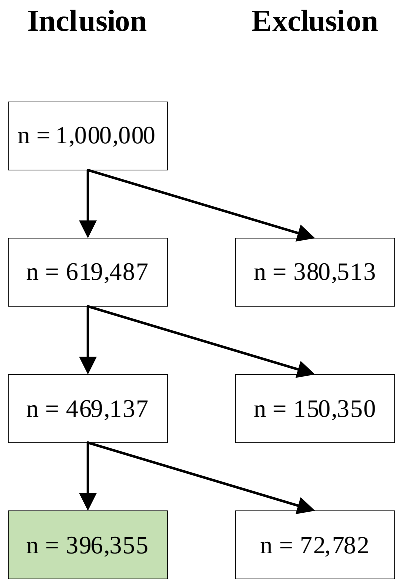

Identify: Random sample of individuals with commercial or MA coverage in 2018 or 2019 who do not have a diagnosis of OUD or treatment for OUD

Continuous enrollment: Exclude cases that did not maintain 12 months continuous enrollment in medical and pharmacy coverage (gaps no longer than 45 days)

Medical encounter: Exclude individuals who did not have at least one medical claim in the inpatient, two claims in the outpatient, or one claim in the ED setting in their index year

At least 18 years of age: Exclude individuals less than 18 years of age in their index year

Notes:
OUD: opioid use disorder
ED: emergency department

© 2021 Anderson KE et al. *JAMA Network Open.*

eFigure 2. Love Plots of Covariate Balance Before and After Propensity Score Matching

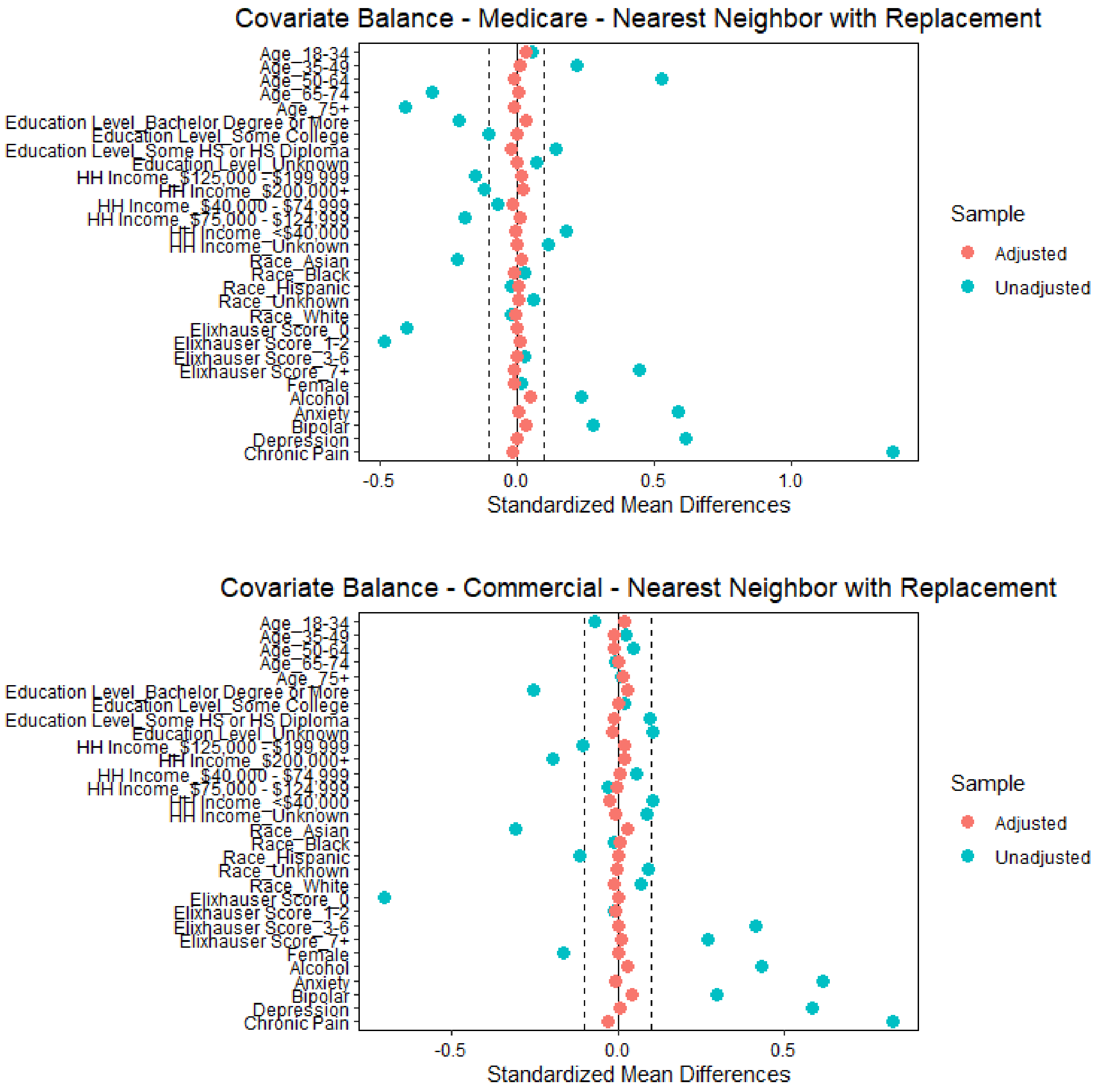

Note: “Unadjusted” shows standardized mean differences before matching and “Adjusted“ shows standardized mean differences after matching.

© 2021 Anderson KE et al. *JAMA Network Open.*

eTable 1. *ICD-10* Codes for Opioid Use Disorder (OUD)

Code	Code Description
F1120	Opioid dependence, uncomplicated
F11220	Opioid dependence with intoxication, uncomplicated
F11221	Opioid dependence with intoxication delirium
F11222	Opioid dependence with intoxication with perceptual disturbance
F11229	Opioid dependence with intoxication, unspecified
F1123	Opioid dependence with withdrawal
F1124	Opioid dependence with opioid-induced mood disorder
F11250	Opioid dependence with opioid-induced psychotic disorder with delusions
F11251	Opioid dependence with opioid-induced psychotic disorder with hallucinations
F11259	Opioid dependence with opioid-induced psychotic disorder, unspecified
F11281	Opioid dependence with opioid-induced sexual dysfunction
F11282	Opioid dependence with opioid-induced sleep disorder
F11288	Opioid dependence with other opioid-induced disorder
F1129	Opioid dependence with unspecified opioid-induced disorder
F1110	Opioid abuse, uncomplicated
F11120	Opioid abuse with intoxication, uncomplicated
F11121	Opioid abuse with intoxication delirium
F11122	Opioid abuse with intoxication with perceptual disturbance
F11129	Opioid abuse with intoxication, unspecified
F1114	Opioid abuse with opioid-induced mood disorder
F11150	Opioid abuse with opioid-induced psychotic disorder with delusions
F11151	Opioid abuse with opioid-induced psychotic disorder with hallucinations
F11159	Opioid abuse with opioid-induced psychotic disorder, unspecified
F11181	Opioid abuse with opioid-induced sexual dysfunction
F11182	Opioid abuse with opioid-induced sleep disorder
F11188	Opioid abuse with other opioid-induced disorder
F1119	Opioid abuse with unspecified opioid-induced disorder
T400X1A	Poisoning by opium, accidental (unintentional), initial encounter
T400X1D	Poisoning by opium, accidental (unintentional), subsequent encounter
T400X1S	Poisoning by opium, accidental (unintentional), sequela
T400X4A	Poisoning by opium, undetermined, initial encounter
T400X4D	Poisoning by opium, undetermined, subsequent encounter
T400X4S	Poisoning by opium, undetermined, sequela
T401X1A	Poisoning by heroin, accidental (unintentional), initial encounter
T401X1D	Poisoning by heroin, accidental (unintentional), subsequent encounter
T401X1S	Poisoning by heroin, accidental (unintentional), sequela
T401X4A	Poisoning by heroin, undetermined, initial encounter
T401X4D	Poisoning by heroin, undetermined, subsequent encounter
T401X4S	Poisoning by heroin, undetermined, sequela
T402X1A	Poisoning by other opioids, accidental (unintentional), initial encounter
T402X1D	Poisoning by other opioids, accidental (unintentional), subsequent encounter
T402X1S	Poisoning by other opioids, accidental (unintentional), sequela
T402X4A	Poisoning by other opioids, undetermined, initial encounter
T402X4D	Poisoning by other opioids, undetermined, subsequent encounter
T402X4S	Poisoning by other opioids, undetermined, sequela
T403X1A	Poisoning by methadone, accidental (unintentional), initial encounter

© 2021 Anderson KE et al. *JAMA Network Open.*

Code	Code Description
T403X1D	Poisoning by methadone, accidental (unintentional), subsequent encounter
T403X1S	Poisoning by methadone, accidental (unintentional), sequela
T403X4A	Poisoning by methadone, undetermined, initial encounter
T403X4D	Poisoning by methadone, undetermined, subsequent encounter
T403X4S	Poisoning by methadone, undetermined, sequela
T404X1A	Poisoning by synthetic narcotics, accidental (unintentional), initial encounter
T404X1D	Poisoning by synthetic narcotics, accidental (unintentional), subsequent encounter
T404X1S	Poisoning by synthetic narcotics, accidental (unintentional), sequela
T404X4A	Poisoning by synthetic narcotics, undetermined, initial encounter
T404X4D	Poisoning by synthetic narcotics, undetermined, subsequent encounter
T404X4S	Poisoning by synthetic narcotics, undetermined, sequela
T40601A	Poisoning by unspecified narcotics, accidental (unintentional), initial encounter
T40601D	Poisoning by unspecified narcotics, accidental (unintentional), subsequent encounter
T40601S	Poisoning by unspecified narcotics, accidental (unintentional), sequela
T40604A	Poisoning by unspecified narcotics, undetermined, initial encounter
T40604D	Poisoning by unspecified narcotics, undetermined, subsequent encounter
T40604S	Poisoning by unspecified narcotics, undetermined, sequela
T40691A	Poisoning by other narcotics, accidental (unintentional), initial encounter
T40691D	Poisoning by other narcotics, accidental (unintentional), subsequent encounter
T40691S	Poisoning by other narcotics, accidental (unintentional), sequela
T40694A	Poisoning by other narcotics, undetermined, initial encounter
T40694D	Poisoning by other narcotics, undetermined, subsequent encounter
T40694S	Poisoning by other narcotics, undetermined, sequela

© 2021 Anderson KE et al. *JAMA Network Open.*

eTable 2. Estimated Probabilities for Quality Measure Performance for Individuals with OUD Compared to Matched Non-OUD Controls, including Estimated Probabilities for Clinical Covariates

	Breast Cancer		Statin Adherence		HbA1c Testing		Mental Health Follow-up		PAH – Chronic[1]		PAH – Diabetes[1]	
	OUD (n=22,217)	Matched Comparators (n=25,919)	OUD (n=2,825)	Matched Comparators (n=5,774)	OUD (n=19,475)	Matched Comparators (n=21,968)	OUD (n=18,145)	Matched Comparators (n=9,833)	OUD (n=79,372)	Matched Comparators (n=79,372)	OUD (n=79,372)	Matched Comparators (n=79,372)
Gender												
Male [Ref.]	---	---	70.5%	77.2%	81.1%	85.3%	44.5%	46.6%	12.8%	10.0%	9.3%	6.6%
Female	55.4%	64.5%	70.1%	76.0%	80.8%	86.3%	46.0%*	56.3%**	11.9%**	9.0%*	6.9%**	5.2%*
Insurance Type												
Commercial [Reference]	50.5%	66.8%	63.7%	72.3%	79.3%	85.0%	50.9%	59.1%	13.3%	9.6%	11.6%	7.7%
Medicare Advantage	56.6%**	64.1%*	71.3%**	77.0%	81.4%**	86.1%	42.3%**	48.9%**	12.1%**	9.4%	7.5%**	5.6%*
Race												
White [Ref.]	54.5%	62.4%	70.9%	77.2%	80.6%	84.9%	46.8%	59.1%	12.3%	9.3%	7.9%	5.6%
Black	58.5%**	67.3%**	66.0%	69.0%**	79.9%	86.0%	45.4%	42.8%**	12.8%	11.0%**	8.8%	7.4%
Asian	54.0%	65.6%	83.0%	77.8%	78.7%	91.8%*	41.0%	47.2%	11.8%	13.2%	10.6%	4.6%
Hispanic	62.1%**	66.7%*	74.2%	74.3%	85.9%**	86.1%	47.5%	43.3%**	10.8%**	9.0%	6.8%	8.2%*
Education												
≤HS Diploma [Ref.]	56.2%	65.5%	68.0%	78.0%	80.5%	85.2%	39.5%	50.7%	12.3%	9.1%	7.9%	5.3%
Some College	56.1%	66.5%	70.4%	75.6%	81.1%	86.7%	45.7%**	48.7%	12.4%	9.9%	7.9%	6.1%
College Degree+	57.0%	71.5%*	75.1%	79.3%	81.9%	85.7%	51.0%**	57.3%	12.2%	9.1%	7.2%	4.8%
Income												
<$40,000 [Ref.]	54.2%	64.8%	71.6%	75.0%	81.3%	86.5%	42.9%	49.8%	12.4%	9.9%	8.0%	6.2%
$40,000-$74,999	59.3%**	67.5%	69.9%	77.9%	81.1%	85.6%	46.8%**	53.2%	11.8%	8.9%	7.1%*	4.9%
$75,000-$124,999	59.7%**	69.3%*	73.9%	80.2%	81.9%	86.5%	53.6%**	55.6%	11.9%	8.9%	6.8%*	5.9%
$125,000-$199,999	60.6%**	68.0%	72.1%	71.0%	80.3%	85.9%	50.6%**	60.5%*	12.0%	9.8%	8.0%	5.4%
$200,000+	64.4%**	73.1%*	68.8%	83.4%	84.0%	89.1%	55.8%**	73.9%**	13.5%	8.6%	8.8%	1.4%*
Elixhauser												

© 2021 Anderson KE et al. *JAMA Network Open.*

Zero (0) [Ref.]	46.6%	62.0%	---	91.3%	60.3%	79.8%	21.2%	32.1%	---	---	---	---
Low (1-2)	52.4%**	64.6%	68.2%[2]	78.3%	78.0%**	84.3%	54.1%**	65.2%*	1.5%[2]	0.7%[2]	---	---
Medium (3-6)	56.9%**	65.4%	72.1%	79.6%	80.9%**	85.5%	49.6%**	55.2%	6.3%**	4.1%**	2.0%[3]	1.4%[3]
High (≥ 7)	55.9%**	63.6%	69.1%	74.8%	82.0%**	86.7%	39.1%*	47.1%	22.0%**	17.4%**	11.8%**	8.8%**
Hypertension												
No [Ref.]	55.9%	66.3%	78.8%	84.0%	68.1%	75.2%	44.7%	43.9%	7.0%	5.2%	7.1%	7.7%
Yes	55.2%	64.0%	81.4%**	86.2%	70.5%	76.7%	45.7%	56.7%**	12.9%**	9.8%**	7.9%	5.7%
Hyperlipidemia												
No [Ref.]	47.3%	57.0%	71.5%	79.1%	68.6%	75.4%	44.3%	54.3%	12.2%	10.0%	7.5%	5.4%
Yes	60.4%**	67.9%**	84.9%**	86.2%**	70.5%	76.7%	46.4%*	50.9%	12.3%	9.3%	8.0%	5.9%
CAD												
No [Ref.]	55.8%	64.7%	81.4%	86.3%	72.5%	74.9%	45.9%	55.5%	10.8%	8.0%	7.0%	4.8%
Yes	52.8%**	64.0%	79.2%**	83.9%	69.9%	76.9%	42.3%**	34.2%**	15.1%**	12.0%**	9.1%**	7.3%**
CVA												
No [Ref.]	55.6%	65.1%	81.0%	85.7%	70.2%	77.6%	45.7%	52.0%	11.9%	9.1%	7.7%	5.7%
Yes	49.1%**	50.9%**	79.1%	88.9%	72.0%	67.7%*	38.3%**	63.8%	17.6%**	14.7%**	10.4%**	7.2%
Asthma												
No [Ref.]	55.1%	64.1%	80.9%	85.9%	69.9%	76.1%	44.9%	48.7%	12.0%	9.5%	7.9%	6.0%
Yes	56.7%	66.5%	80.9%	85.4%	72.7%	79.5%	47.1%*	65.0%**	13.4%**	9.1%	7.8%	4.9%
COPD												
No [Ref.]	56.8%	66.4%	81.3%	86.1%	70.9%	76.7%	45.8%	52.8%	8.9%	7.0%	8.3%	6.0%
Yes	52.4%**	64.0%**	80.0%*	85.1%	69.6%	76.6%	44.3%	51.9%	16.3%**	13.0%**	7.4%*	5.5%
Arthritis												
No [Ref.]	51.0%	62.3%	79.5%	86.0%	70.9%	74.2%	45.6%	53.2%	12.5%	9.4%	8.9%	5.9%
Yes	57.1%**	66.0%**	81.6%**	85.7%	70.0%	78.1%	45.1%	51.6%	12.2%	9.4%	7.5%**	5.7%
Diabetes												
No [Ref.]	55.5%	65.1%	46.5%	46.3%	71.3%	76.3%	44.1%	54.6%	11.5%	8.9%	---	---
Yes	55.1%	63.7%	87.2%**	91.5%**	69.6%	76.9%	48.5%**	48.6%*	13.0%**	9.8%*	7.9%	5.8%
Depression												
No [Ref.]	55.5%	65.1%	80.5%	86.0%	71.5%	77.6%	34.2%	26.6%	12.7%	9.9%	7.7%	5.6%
Yes	55.4%	64.1%	81.3%	85.7%	68.9%	67.7%	46.2%**	54.1%**	12.0%**	9.1%	8.1%	5.9%
Anxiety Disorder												
No [Ref.]	57.1%	65.9%	82.3%	86.5%	73.0%	76.3%	32.6%	41.2%	11.0%	8.2%	7.0%	5.0%
Yes	53.8%**	63.3%*	79.5%**	85.2%	66.8%**	77.1%	47.0%**	53.5%**	13.4%**	10.6%**	8.8%**	6.6%*
Bipolar Disorder												
No [Ref.]	55.8%	64.4%	81.0%	85.8%	70.4%	76.7%	42.3%	47.8%	12.2%	9.2%	7.7%	5.7%
Yes	52.3%**	65.7%	80.6%	86.1%	70.2%	76.0%	47.4%**	55.3%**	12.8%	11.1%*	8.7%	6.1%

© 2021 Anderson KE et al. *JAMA Network Open.*

Hepatitis C												
No [Ref.]	55.5%	64.5%	81.0%	85.7%	70.8%	76.9%	45.4%	52.9%	12.2%	9.4%	7.8%	5.7%
Yes	53.1%	66.5%	79.1%	90.9%	59.9%*	62.6%	44.9%	41.3%	13.3%*	11.0%	9.9%**	10.7%
HIV/AIDS												
No [Ref.]	55.4%	64.5%	80.9%	85.8%	70.3%	76.6%	45.3%	51.1%	12.3%	9.4%	7.9%	5.8%
Yes	49.7%	73.1%	79.0%	86.7%	77.8%	81.1%	46.9%	41.3%**	10.3%	8.9%	6.9%	9.0%
Alcohol Use Disorder												
No [Ref.]	55.8%	65.0%	81.4%	86.4%	70.9%	77.6%	43.8%	54.1%	12.0%	9.3%	7.8%	5.6%
Yes	49.0%**	57.1%*	77.0%**	79.3%*	62.6%*	64.1%*	47.5%**	50.0%	14.7%**	10.5%	8.8%	7.8%
Chronic Pain												
No [Ref.]	57.2%	65.9%	81.8%	86.5%	69.3%	78.3%	45.2%	56.9%	11.3%	9.0%	6.7%	5.1%
Yes	55.0%**	64.2%	80.7%	85.7%	70.7%	76.2%	45.4%	50.5%**	12.5%**	9.5%	8.2%**	5.9%

[1] For the PAH (potentially avoidable hospitalization) indicators, a lower indicator score indicates better quality of care. For all other indicators, a higher score indicates better quality of care.

[2] Because an Elixhauser score of zero was omitted from the chronic composite indicator regression, we treated the category of Low (1-2) as the reference group.

[3] Because an Elixhauser score of zero and a low Elixhauser score (1-2) were omitted from the chronic composite indicator regression, we treated the category of Medium (3-6) as the reference group.

“Unknown” response options for race, education, and income were included in the model but not reported in the table.

*p≤0.05, **p≤0.01 statistically significant difference between the reference group (first row) and the comparator category.

© 2021 Anderson KE et al. *JAMA Network Open.*
